# COIMMR: a computational framework to reveal the contribution of herbal ingredients against human cancer via immune microenvironment and metabolic reprogramming

**DOI:** 10.1093/bib/bbad346

**Published:** 2023-10-06

**Authors:** Saisai Tian, Yanan Li, Jia Xu, Lijun Zhang, Jinbo Zhang, Jinyuan Lu, Xike Xu, Xin Luan, Jing Zhao, Weidong Zhang

**Affiliations:** College of Pharmacy, Ningxia Medical University, Yinchuan 750004, China; School of Pharmacy, Second Military Medical University, Shanghai, 200433, China; College of Pharmacy, Ningxia Medical University, Yinchuan 750004, China; School of Pharmacy, Second Military Medical University, Shanghai, 200433, China; School of Pharmacy, Second Military Medical University, Shanghai, 200433, China; College of Pharmacy, Henan University, Kaifeng 475000, China; The Research Center for Traditional Chinese Medicine, Shanghai Institute of Infectious Diseases and Biosafety, Institute of Interdisciplinary Integrative Medicine; Research, Shanghai University of Traditional Chinese Medicine, Shanghai, China Department of Pharmacy, Tianjin Rehabilitation Center of Joint Logistics Support Force, Tianjin, 300110, China; College of Pharmacy, Anhui University of Chinese Medicine, Anhui 230012, China; School of Pharmacy, Second Military Medical University, Shanghai, 200433, China; The Research Center for Traditional Chinese Medicine, Shanghai Institute of Infectious Diseases and Biosafety, Institute of Interdisciplinary Integrative Medicine; The Research Center for Traditional Chinese Medicine, Shanghai Institute of Infectious Diseases and Biosafety, Institute of Interdisciplinary Integrative Medicine; College of Pharmacy, Ningxia Medical University, Yinchuan 750004, China; School of Pharmacy, Second Military Medical University, Shanghai, 200433, China; The Research Center for Traditional Chinese Medicine, Shanghai Institute of Infectious Diseases and Biosafety, Institute of Interdisciplinary Integrative Medicine

**Keywords:** immune microenvironment, metabolic reprogramming, COIMMR, new computational framework, herbal ingredients

## Abstract

Immune evasion and metabolism reprogramming have been regarded as two vital hallmarks of the mechanism of carcinogenesis. Thus, targeting the immune microenvironment and the reprogrammed metabolic processes will aid in developing novel anti-cancer drugs. In recent decades, herbal medicine has been widely utilized to treat cancer through the modulation of the immune microenvironment and reprogrammed metabolic processes. However, labor-based herbal ingredient screening is time consuming, laborious and costly. Luckily, some computational approaches have been proposed to screen candidates for drug discovery rapidly. Yet, it has been challenging to develop methods to screen drug candidates exclusively targeting specific pathways, especially for herbal ingredients which exert anti-cancer effects by multiple targets, multiple pathways and synergistic ways. Meanwhile, currently employed approaches cannot quantify the contribution of the specific pathway to the overall curative effect of herbal ingredients. Hence, to address this problem, this study proposes a new computational framework to infer the **c**ontribution **o**f the **i**mmune **m**icroenvironment and **m**etabolic **r**eprogramming (COIMMR) in herbal ingredients against human cancer and specifically screen herbal ingredients targeting the immune microenvironment and metabolic reprogramming. Finally, COIMMR was applied to identify isoliquiritigenin that specifically regulates the T cells in stomach adenocarcinoma and cephaelin hydrochloride that specifically targets metabolic reprogramming in low-grade glioma. The *in silico* results were further verified using *in vitro* experiments. Taken together, our approach opens new possibilities for repositioning drugs targeting immune and metabolic dysfunction in human cancer and provides new insights for drug development in other diseases. COIMMR is available at https://github.com/LYN2323/COIMMR.

## INTRODUCTION

Cancer, a major public health concernworldwide, accounts for >70% of all deaths worldwide[1, 2]. During carcinogenesis, cancer cells gradually acquire malignant phenotypes, including uncontrolled replicative potential, enhanced energy metabolism and immune escape, which contribute to poor prognosis and consequently decrease the survival rate [3–5]. Notably, immune evasion and metabolism reprogramming have been regarded as two vital hallmarks of cancer development [6]. Thus, targeting the immune microenvironment and the reprogrammed metabolic processes is a promising strategy for developing novel anti-cancer drugs. In recent decades, herbal medicine, a mainstream form of complementary and alternative medicine, has been widely used to treat cancer worldwide [7, 8]. Many herbal ingredients exert anti-cancer effects, such as inhibiting the development, proliferation, angiogenesis and metastasis of human cancer [9, 10] by multiple targets and multiple pathways [11, 12]. In particular, many herbal ingredients modify the tumor microenvironment, potentiate the function of immune cells [13, 14] and regulate metabolic reprogramming [15]. For example, Astragalus polysaccharide, which is the main component of qi tonifying herb *Astragalus membranaceus*, inhibited Foxp3 mRNA expression through cytokine and matrix repair, suppressed SDF-1's recruitment of regulatory Treg cells to the microenvironment of liver cancer and mitigated the immunosuppressive effect of Treg cells [16, 17]. Chlorogenic acid, a phenolic acid derived from *Eucommia ulmoides* Oliv*.*, regulates the glucose metabolism in HepG2 cells [18] and inhibits the hypoxia-inducible factor-1α and vascular endothelial growth factor expression in DU145 [19] and A549 cells [20] to suppress tumor angiogenesis and progression.

However, labor-based screening is not suitable for identifying candidates specifically targeting the immune microenvironment and metabolic reprogramming, as it is a time-consuming and costly process [21, 22]. Some computational approaches have been proposed to rapidly screen candidate compounds [23–26]. For example, Wang *et al*. proposed a tumor immunological phenotype signature-based computational method to identify novel targeted immunotherapies [27]. Unluckily, these approaches cannot be used to quantify the contribution of specific pathways to the overall curative effect (OCE) of a drug.

Hence, to address this problem, this study proposed a new computational framework, contribution of the immune microenvironment and metabolic reprogramming (COIMMR), to reveal the contribution of the regulatory effects of herbal ingredients on the immune microenvironment and metabolic reprogramming in their anti-human cancer effects and to further screen the herbal ingredients specifically targeting the immune microenvironment and reprogrammed metabolic processes. The data of 30 cancers were obtained from The Cancer Genome Atlas (TCGA), while those of 496 herbal ingredients were obtained from the Integrated Traditional Chinese Medicine (ITCM) database, which is the largest herbal ingredient-based pharmacotranscriptomic platform built by our team [28]. Then, the high-quality immune-specific signatures (ISs) and metabolic-specific signatures (MSs) were collected, and the normalized enrichment score (NES) of each signature for each human cancer and herbal ingredient was qualified. Furthermore, the immunological landscape or metabolic landscape of human cancer and herbal ingredients were constructed. Finally, the contribution of the regulatory effects of herbal ingredients on the immune microenvironment and metabolic reprogramming to their anti-human cancer activities was quantified.

In total, 14 880 possible interactions between herbal ingredients and human cancer were examined. Among the immunological interactions, 12 247 significant interactions were identified. Of these, 6356 interactions were negatively correlated, indicating that the herbal ingredients might exert therapeutic effects on human cancer via immune regulation. Among metabolic interactions, 1049 significant interactions were identified. Of these, 493 interactions were negatively correlated, indicating that these herbal ingredients might exert therapeutic effects on human cancer via metabolic regulation. Furthermore, isoliquiritigenin (ISL) and cephaelin hydrochloride (CH) that target the T cells in stomach adenocarcinoma (STAD) and metabolic reprogramming in low-grade glioma (LGG), respectively, were identified to demonstrate the power of this method.

The findings of this study indicate that this novel approach can quantify the contribution of herbal ingredients against human cancer via specific pathways or biological function. In addition, this approach also provides novel insights for drug discovery. COIMMR is free and available via GitHub at https://github.com/LYN2323/COIMMR.

## MATERIALS AND METHODS

### Data collection and pre-processing

Cancer gene expression profiles were downloaded from the TCGA database, while those of the corresponding normal tissue gene expression profiles were obtained from the GTEx database. The values were standardized by TPM (transcripts per kilobase million) and expressed as log2 (TPM + 0.001) for subsequent analysis. After excluding cancer types lacking control samples, datasets comprising 30 human cancer gene expression profiles (ACC, BLCA, LGG, BRCA, CESC, COAD, DLBC, ESCA, GBM, HNSC, KICH, KIRC, KIRP, LIHC, LUAD, LUSC, OV, PAAD, PCPG, PRAD, SARC, SKCM, STAD, THYM, THCA, UCS, UCEC, CHOL, LAML and READ) and corresponding normal tissue gene expression profiles were selected for further analysis. Herbal ingredient-related transcriptomic data were obtained from ITCM, which included 496 active ingredients from unified high-throughput experiments developed by our team [28]. In addition, another independent pharmacotranscriptomic dataset GSE85871, which included 102 herbal ingredients from unified microarray experiment, were downloaded to enhance the robustness of our model. Detailed data processing are provided in previous studies [28, 29].

To avoid the result of pleiotropy caused by other pathways, ISs and MSs were manually curated from the high-quality literatures for this research. High-quality ISs were manually curated from the latest study [30] using a two-step process. First, the differences between two steady-state immune profiles were analyzed to obtain 304 differential gene expressions. Second, a set of 608 ISs was defined by selecting the top 250 genes up-regulated and the top 250 genes down-regulated in each immune cell state transition (Supplementary Table S1). Meanwhile, 114 MSs also were collected from the relevant high-quality literature [31]. Given that some MSs with little overlap with background genes could not be enriched for analysis, they were removed from the analysis. A final set of 69 MSs were used for further analysis (Supplementary Table S2). In addition, each cancer-specific signature (CSS, the top 250 up-regulated genes and the top 250 down-regulated genes) was obtained using differential expression analysis with the ‘limma’ package (Supplementary Figure S1, Supplementary Table S3). The detailed criteria of signature size are listed in Supplementary Material.

### Construction of immunological landscape and metabolic landscape

For each cancer and herbal ingredient, gene set enrichment analysis (GSEA) was performed to calculate the NES values of corresponding ISs and MSs to establish the immunological and metabolic landscapes. Positive and negative scores indicate the up-regulation and down-regulation, respectively, of the signatures related to immune status change and metabolic patterns in each cancer or herb ingredient. To qualify the immunomodulatory and metabolic modulating capacities of herbal ingredients against cancer, the similarity score, also named as immunological similarity score (*I*_score_) or metabolic similarity score (*M*_score_), were defined using the person correlation method. Based on the similarity score, the therapeutic relationship between human cancer and ingredients was identified. The similarity score <0 means therapeutic relationship, with smaller scores indicating greater likelihood of treating cancer.

To assess the significance of the therapeutic relationship, 608 ISs were first generated randomly from 20 300 background genes, each IS consisting of 250 genes. Subsequently, the randomly generated ISs were subjected to GSEA with the expression profiles of cancer and herbal ingredients, and finally the two NES vectors were correlated to obtain the *I*_score_. The above process was repeated 1000 times to obtain the NULL distribution of *I*_score_. The previously calculated immune similarity scores were compared with the NULL distribution to calculate *P*-values and FDR. The permutation test evaluation for *M*_score_ is the same as for *I*_score_.

### Reverse GSEA of CSS in the herbal ingredient gene expression profiles

Genes linked to the same disease were typically closely correlated to each other, and the expression and correlation patterns of these genes were similar [32]. Hence, changes in the gene expression pattern can indicate a pathological state. If a drug mitigates this change, it can potentially exert therapeutic effects on the disease. Briefly, if the gene expression pattern induced by a drug (drug signatures) contrasts with that induced by a disease (disease signatures), the drug might exhibit potentially therapeutic effects on the disease [33–35].

G was assumed to be a group of genes known to be associated with a disease. Each matrix of gene expression values (X_1_, X_2_, … , X_N_) had N genes whose expression levels were determined using RNA sequencing. CSSs are identified by selecting the top 250 up-regulated genes and the top 250 down-regulated genes. The same method as that of GSEA was used to determine if herbal ingredients significantly enriched the expression of CSSs. This process was termed RGSEA to distinguish it from GSEA (Supplementary Figure S2). NES_up_ and NES_down_ were calculated for the up-regulated and down-regulated gene sets, respectively. Ideally, the NES of up-regulated CSS of each cancer in a drug is less than 0, and the NES of down-regulated CSS in a drug is greater than 0. The OCE score (OCES) of each herbal ingredient against each cancer is as follows:

OCES = NES_up_ − NES_down_.

Finally, 1000 times permutation testing were performed, and 1000 NES values constitute a NULL distribution. In addition, the FDR values are computed.

### The contribution of the immune microenvironment and reprogrammed metabolic processes

In the way above, a 30-dimension vector of *I*_score_, *M*_score_ and OCES for each herbal ingredient was produced. The correlations between OCES and *I*_score_ or *M*_score_ was determined by Pearson’s correlation analysis to quantify the contribution of the regulatory effects of herbal ingredients on the immune microenvironment and reprogrammed metabolic processes to their anti-human cancer activities. The scores were directly proportional to the degree of contribution, with larger scores indicating greater contribution of treating cancer.

### Validation the power of COIMMR using *in vitro* experiment

Several case studies were performed todemonstrate its strong power of COIMMR. In brief, we applied COIMMR to identify ISL that specifically regulates the T cellsin STAD and CH that specifically targets metabolic reprogramming in LGG. Furthermore, we performed cell viability assay, co-culture experiments and western blotting to verify the computational results. The detailed experimental procedure is listed in Supplementary Material.

## RESULTS

### A novel method to infer the contribution of immune microenvironment and metabolic reprogramming

**Figure 1:**
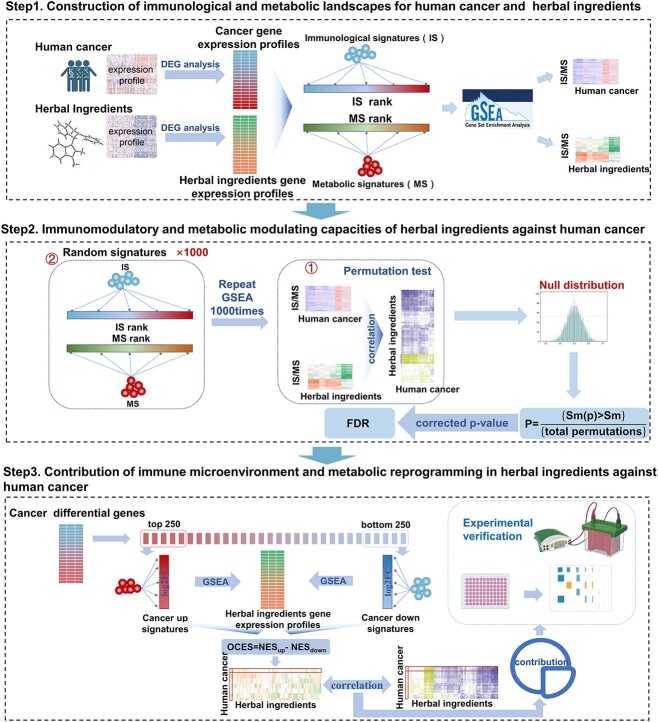
Schematic diagram of COIMMR.

The schematic for COIMMR is shown in Figure 1, and the technical details are provided in Methods. COIMMR can be used comprehensively of any established gene sets, such as the signatures of immune microenvironment and metabolic reprogramming. In this method, the ISs and MSs were collected to construct the immunological and metabolic landscape for human cancer and herbal ingredients. Next, correlation analysis was performed to qualify the immunomodulatory and metabolic regulation capacities of herbal ingredients on human cancer. Permutation testing was utilized to evaluate the statistical significance of each herbal ingredient associated with each human cancer at the immune microenvironment and metabolic reprogramming level. The following formula was used to perform 1000 times permutation testing:


$$ P=\frac{\left\{ Sm(p)> Sm\right\}}{\left\{\mathrm{total}\ \mathrm{permutations}\right\}}. $$

A nominal *P* was calculated by counting the number of *I*_score_ or *M*_score_ of the NULL distribution (*Sm(p)*) obtained from the permutation test greater than the original similarity score (*Sm*). Furthermore, the overall therapeutic capacity of each herbal ingredient against human cancer was determined using RGSEA. Finally, the contribution of the regulatory effects of herbal ingredients on the immune microenvironment and metabolic reprogramming to their anti-human cancer activities was determined by calculating the Pearson correlation between cancer-specific OCES and their *I*_score_ or *M*_score_.

### Depicting immunological and metabolic landscape for human cancer

**Figure 2:**
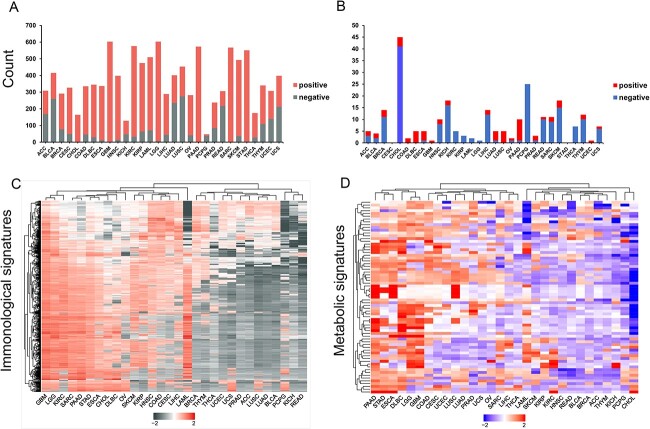
The associations between human cancer and immunological changes or metabolic pathway. (**A**) The numbers of the immunological signatures that were significantly positively or negatively affected in each human cancer. (**B**) The numbers of metabolic signatures that were significantly positively or negatively affected in each human cancer. (**C**) Clustered heatmap of immunological landscape of human cancer organized according to the NES values. (**D**) Clustered heatmap of metabolic landscape of human cancer organized according to the NES values. The full names of abbreviations can be found in the abbreviation section.

With the GSEA approach, we systematically constructed relationships between 30 cancers and 608 ISs or 69 MSs with FDR ≤0.05. As shown in Figure 2A, more than half of the immune-related changes were significantly enriched in most human cancers (Supplementary Table S4), which is consistent with the deep involvement of the immune microenvironment in carcinogenesis. For example, immune responses against cancer are induced by immune cells such as T cells, natural killer (NK) cells, macrophages and effector T cells, and enter an “exhausted” state with chronic antigen stimulation and inflammation within the tumor microenvironment, which contributes to T cell dysfunction and tumor formation [36]. NK cells primarily mediate the cytotoxic response against tumors by releasing perforin and granzymes to induce cell death [37]. In addition to releasing anti-inflammatory cytokines and suppressing the immune responses against tumor cells, the M2-polarized macrophages promote angiogenesis and matrix remodeling to induce the spread of the tumor and its metastasis [38]. It is worth noting that GBM, LGG, KIRC, PAAD, SARC, STAD and LAML involved at least 80% of ISs (Supplementary Table S4). In addition, based on the number of significantly enriched signatures, 42 ISs were significantly associated with almost all human cancers (Supplementary Table S5), which mainly includes changes in the status of T cell, followed by the B cells, NK cells, dendritic cells, stromal cells and monocytes.

In terms of metabolic reprogramming, half of the cancers significantly enriched seven MSs. Notably, among human cancers, CHOL is enriched for 45 metabolic pathways (Figure 2B, Supplementary Table S6). For example, in the tryptophan metabolic pathway, tryptophan undergoes a two-step enzymatic reaction (hydroxylation and decarboxylation) to produce serotonin. It has been shown that serotonin administration increases the growth of cholangiocarcinoma cells *in vitro*, while inhibition of serotonin synthesis suppresses tumor cell growth *in vitro* and *in vivo* [39]. Besides, the number of significantly enriched metabolic pathways was counted. Tyrosine metabolism was associated with more than half of the cancers (Supplementary Table S7). In the last years, several tyrosine kinases have been identified as molecular targets for inhibiting tumor growth. Consequently, various tyrosine kinase inhibitors have been developed [40, 41]. It has been reported that tyrosine kinase inhibitors typically inhibit the active site of tyrosine kinases, blocking the activation of downstream signaling pathways involved in cell proliferation or angiogenesis. In addition, tyrosine kinase inhibitors also can promote metabolic reprogramming in human cancer [42].

Finally, the NES values were visualized using a heatmap. Different cancers were organized by unsupervised hierarchical clustering. Human cancers, such as SKCM, KIRP, HNSC, COAD, CESC and LIHC, were clustered into one group (Figure 2C). Their NES values of these cancers were mostly positive, indicating that they might share similar mechanisms to induce changes in immune status. In contrast, PCPG, KICH and READ were clustered in one group with mostly negative NES values, suggesting that their regulatory effects on the immune status may be contrast with those of SKCM, KIRP, LIHC, HNSC, CESC and COAD (Figure 2C). Similarly, BLCA, BRCA, ACC and THYM exhibit consistent metabolic similarities (Figure 2D). The mechanisms of metabolic regulation in PAAD, STAD, ESCA, DLBC and LGG are different from those in BLCA, BRCA, ACC and THYM (Figure 2D). These findings suggest the presence of complex and diverse immunological processes and metabolic regulatory mechanisms in human cancer.

### Depicting immunological and metabolic landscape for herbal ingredients

**Figure 3:**
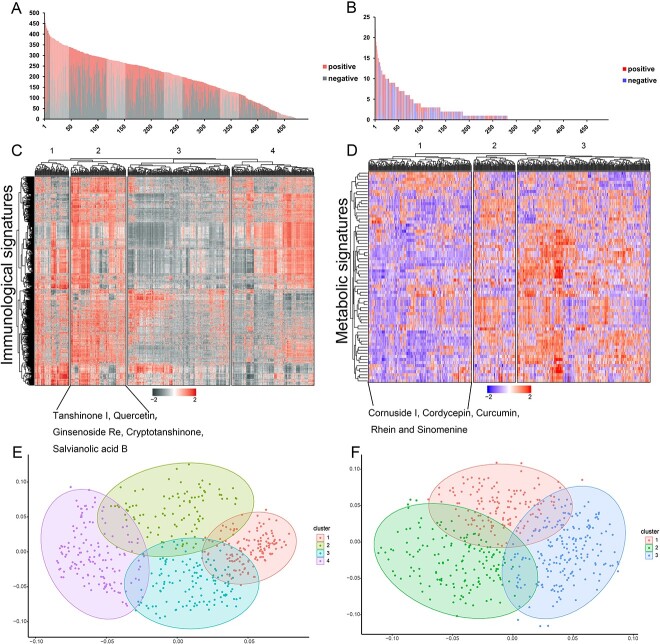
The associations between herbal ingredients and immunological changes or metabolic pathway. (**A**, **B**) The numbers of the immunological (**A**) or metabolic (**B**) signatures that were significantly positively or negatively affected by each herbal ingredient. The full names of the horizontal coordinates of the distribution are provided in Supplementary Table S8 and Supplementary Table S10. (**C**, **E**) Clustered heatmap and PCA diagram of immunological landscapes of herbal ingredients organized according to the NES values. (**D**, **F**) Clustered heatmap and PCA diagram of metabolic landscapes of herbal ingredients organized according to the NES values.

Similar to the construction of the immunological landscape and metabolic landscape of cancer, the immunological and metabolic landscapes of herbal ingredients also were constructed by calculating the NES values for each herbal ingredient. As shown in Figure 3A, more than half of the herbal ingredients significantly affected >200 immune status changes (Supplementary Table S8), which suggests that many herbal ingredients, such as dioscin, polyphyllin I, genkwanin, ginsenoside Rb3 and isofraxidin, may play a protective role by regulating the immune microenvironment. For example, it has been observed that dioscin induced macrophage M2-to-M1 phenotype transition *in vitro* and inhibited IL-10 secretion, and it may act as a new anti-tumor agent by down-regulating STAT3 and JNK signaling pathways in macrophages *in vitro* [43]. Polyphyllin I demonstrated effective amelioration of synovial inflammation in the ankle joint of CIA mice while suppressing NF-κB-mediated production of pro-inflammatory effectors in activated macrophages [44]. Also, genkwanin may exert anti-tumor activity by enhancing host immunity and reducing inflammatory cytokine levels [45]. Equally, ginsenoside Rb3 ameliorated *Porphyromonas gingivalis* LPS-induced inflammation by inhibiting the MAPK/AKT/NF-κB signaling pathways and attenuated alveolar bone resorption in experimental periodontitis rats [46]. In addition, studies have shown that isofraxidin possesses significant analgesic and anti-inflammatory activities that may be mediated through the regulation of pro-inflammatory cytokines, TNF-α and the phosphorylation of p38 and ERK1/2 [47]. Similarly, the number of significantly enriched herbal ingredient-related ISs was counted. The top two ISs significantly associated with the herbal ingredients were T cell-related and stem cell-related state changes (Supplementary Table S9). In terms of metabolism, 57% of herbal ingredients, including demethoxycurcumin, indirubin, loganin, ethyl caffeate and isorhamnetin, were significantly associated with at least one metabolic pathway (Figure 3B, Supplementary Table S10). Then, the number of metabolic pathways significantly associated with herbal ingredients was counted. Noticeably, the cholesterol metabolic pathway was significantly involved in the anti-cancer efficacy of the most herbal ingredients (Supplementary Table S11). Cholesterol biosynthesis plays a key role in the maintenance of tumor stem cells and contributes significantly to cancer progression, including cell proliferation, migration and invasion [48, 49]. In addition, cholesterol depletion or transport blockage impedes the growth and invasion of various cancers [50].

Unsupervised hierarchical clustering was performed with herbal ingredients, which was classified into four classes based on the immune status and three classes based on the metabolic pathways (Figure 3C–F). It has been reported that herbal ingredients clustered into the same class may share similar immune regulatory mechanisms or metabolic mechanisms [29, 51, 52]. For example, tanshinone I, quercetin, ginsenoside Re, cryptotanshinone and salvianolic acid B were clustered together (Figure 3C), which can exert immune effects by inhibiting the pro-inflammatory cytokine TNF-α [53–57]. In the metabolic landscape, herbal ingredients, such as cornuside I, cordycepin, curcumin, rhein and sinomenine, were in the same group with mostly negative NES values, indicating that they may regulate metabolic pathways through similar mechanisms (Figure 3D). Literature research has found that these herbal ingredients can induce apoptosis in cancer cells and reduce apoptosis in normal functioning cells by affecting mitochondrial energy metabolism [58–62].

### Qualifying immunomodulatory and metabolic modulating capacities of herbal ingredients against human cancer

**Figure 4:**
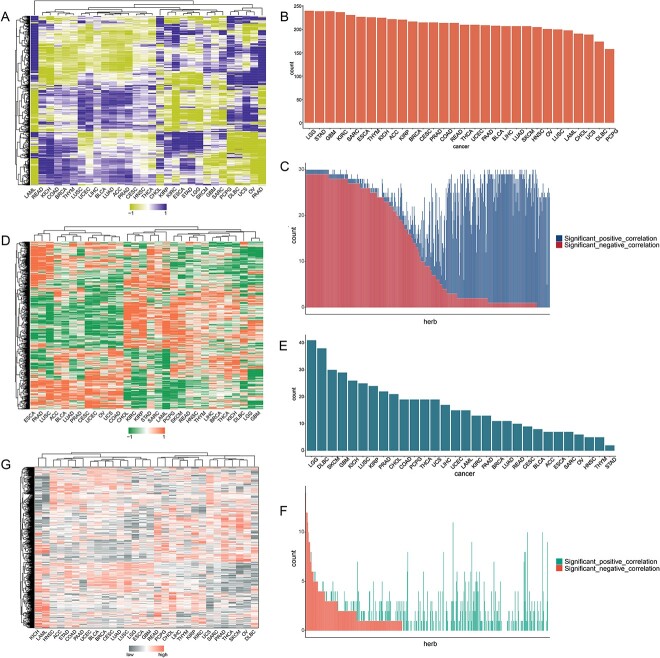
Immunomodulatory and metabolic modulating capacities of herbal ingredients against human cancer. (**A**) Heatmap of herbal ingredients–human cancer associations qualified by *I*_score_. (**D**) Heatmap of herbal ingredients–human cancer associations qualified by *M*_score_. (**G**) Heatmap of OCE for herbal ingredients against cancer qualified by OCES. (**B**, **E**) Number of human cancers that were significantly negatively with herbal ingredients based on immune changes (**B**) and metabolic pathways (**E**). (**C**, **F**) Number of herbal ingredients that were significantly negatively and significantly positively with each human cancer based on immune changes (C) and metabolic pathways (F). The full names of the horizontal coordinates of the distribution can be found in Supplementary Tables S13 and S15.

Based on the immunological landscape or metabolic landscape of cancers and herbal ingredients, the correlation between each cancer and each herbal ingredient was established. In terms of immune modulation, the 14 880 corresponding correlations between herbal ingredients and human cancer were identified. Of these, 12 247 were significant and 6356 exhibited negative coefficients, indicating a therapeutic association (Figure 4A). Each cancer had >158 significant therapeutic associations, with the top three cancers being LGG, STAD and GBM (Figure 4B, Supplementary Table S12). Of the 496 herbal ingredients, 47 herbal ingredients were the most closely related to the cancer therapy, and they could potentially revert the progression of almost all human cancers (Figure 4C, Supplementary Table S13). Hence, these herbal ingredients, such as 6-gingerol, cordycepin, platycodin D, linolenic acid and ISL, were considered to exert the most potent therapeutic effects on human cancer. For example, 6-gingerol treatment of tumor-bearing mice caused massive infiltration of CD4^+^ and CD8^+^ T cells and B220^+^ B cells, which inhibited tumor growth in mice [63]. Cordycepin can significantly reduce the production of IL-17A *in vitro* and *in vivo*, and reduce the expression of its downstream signaling molecules to enhance tumor killing effect and reduce PD-L1 expression [64].

In terms of metabolic modulation, 14 880 correspondingrelationships were also identified. Of these, 1049 were significant with 493 exhibiting significantly negative correlations, indicating a therapeutic relationship (Figure 4D). Notably, LGG, DLBC and SKCM were the top three human cancers with the highest number of therapeutic correlations with herbal ingredients (Figure 4E, Supplementary Table S14), suggesting that metabolic reprogramming plays an important role in the development of these cancers. Among the 496 herbal ingredients, 196 were significantly negatively associated with at least one cancer (Figure 4F, Supplementary Table S15).

### The contribution of the regulatory effects of herbal ingredients on the immune microenvironment and reprogrammed metabolic processes in OCE

**Figure 5:**
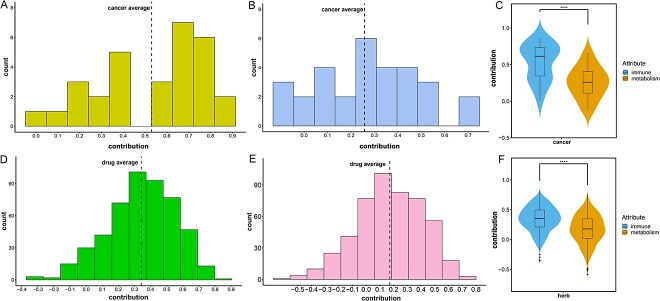
The contribution of immune microenvironment and metabolic reprogramming. (**A**, **B**) The number distribution of the contribution of the immune microenvironment (**A**) and metabolic reprogramming (**B**) in cancer development. (**D**, **E**) The number distribution of the contribution for the regulating immune microenvironment (**D**) and metabolic reprogramming (**E**) in the anti-cancer efficacy of each herbal ingredient. (**C**, **F**) Violin plots of the contribution of immune microenvironment and metabolic reprogramming in cancer (**C**) and herbal ingredients (**F**). Statistical significance: ^*^^*^^*^^*^P < 0.0001.

The OCES of herbal ingredients against human cancer was calculated using RGSEA (Figure 4G). Then, the contribution of the regulatory effects of herbal ingredients on the immune microenvironment and metabolic reprogramming on their anti-human cancer activities was quantified by examining the correlation between OCES and *I*_score_ or *M*_score_. In this study, the average contribution for human cancer based on immune microenvironment was 0.53 (Figure 5A). This finding suggests that modifications in the immune microenvironment are closely correlated with the development of cancer and contribute to a large part of cancer progression. It also might indicate that cancers may likely be treated by the immunomodulatory effects of drugs. The mean correlation coefficient for human cancers in metabolic reprogramming was 0.26 (Figure 5B). This indicates that metabolic reprogramming also has a certain role in the emergence and spread of cancer and cancer can be ameliorated by the metabolic modulating ability of some drugs. Noticeably, the contribution of the immune system to the pathophysiology of cancer is higher than that of metabolic reprogramming as shown in Figure 5C. In other words, the immune system may be more profoundly involved in the pathophysiology of cancer than metabolic reprogramming.

The average contribution of herbal ingredients based on the immune microenvironment was 0.34, while the average contribution of herbal ingredients was 0.17 in terms of metabolic reprogramming (Figure 5D and E), indicating that herbal ingredients is more of a therapeutic effect through immunity. Furthermore, the contribution of immunomodulatory and metabolic modulating abilities of herbal ingredients were qualified and the contribution of immunomodulatory ability was higher than that of metabolic pathway-modulating ability (Figure 5F). Hence, these results may indicate that herbal ingredients exerted a higher modulatory effect on the immune microenvironment than on metabolic reprogramming for their therapeutic effects on cancer.

To further verify the robustness and generalizability of the computational framework, the performance of the computational model was evaluated in the independent pharmacotranscriptomic dataset GSE85871, which included 102 herbal ingredients from unified microarray experiment [29]. We used the same computational formula to calculate the contribution of the regulatory effects of herbal ingredients against human cancer. A similar result was observed that the contribution of the immune system to the pathophysiology of cancer is higher than that of metabolic reprogramming (Supplementary Figure S3). Meanwhile, the contribution of immunomodulatory ability of herbal ingredients was significantly higher than that of metabolic pathway-modulating ability of herbal ingredients (*P* < 0.01) (Supplementary Figure S3). Hence, these results further confirmed the reliability and generalizability of our model.

In summary, these findings indicated that the developmental process of most human cancers might be reversed mainly through the immunomodulatory effects of herbal ingredients.

### Identification of ISL against STAD and CH against in LGG using COIMMR

Through the above analysis, we can identify some representative cancers and herbal ingredients. As shown in Figure 4B, LGG, STAD and GBM were the top three human cancers closely correlated with the immune microenvironment, indicating that these cancers may potentially be treated through immunomodulation using various herbal ingredients. As shown in Figure 2A and B, we can clearly see that the number of immune dysregulated signatures in STAD is >500, while significantly regulated metabolic signatures are very limited. These results suggest that immune dysregulation may be a key factor in the progression of STAD. Hence, STAD was selected as a representative cancer for the modulation of the immune microenvironment. As shown in Figure 4E, LGG was the top-ranked cancer closely correlated with metabolic reprogramming. Hence, LGG was selected as a representative cancer for the modulation of metabolic reprogramming. Then, the herbal ingredients with the most significant effect on STAD and LGG were identified.

The herbal ingredients with significant therapeutic effects on LGG and STAD were selected as follows: (1) the *I*_score_/*M*_score_ of the herbal ingredients with therapeutic correlations with LGG and STAD were ranked from smallest to largest; (2) the NES values of the herbal ingredients with NES <0 of the overall therapeutic effects were ranked and (3) the contribution of the regulatory effects of herbal ingredients on the immune microenvironment and reprogrammed metabolic processes to their anti-cancer efficacy was ranked. In all three steps, the top 20% of herbal ingredients were selected. Finally, nine herbal ingredients (camphor, liguiritigenin-7-*O*-d-apiosyl-4′-*O*-d-glucoside, corydaline, glycyrrhetinic acid, cordycepin, hupehenine, paeoniflorin, isoliquiritin, ISL) with significant immunotherapeutic effects in STAD (supplementary S16) and three herbal ingredients (CH, tenacissoside H, ginsenoside F2) with significant metabolic pathway-modulating effects in LGG (supplementary S17) were identified. Literature review revealed that limited studies have examined the growth-inhibitory effects of ISL and CH against STAD and LGG, respectively. Therefore, ISL and CH were subjected to follow-up experimental verification.

### ISL and CH inhibit proliferation of MGC803 cells and SW1783 cells

**Figure 6:**
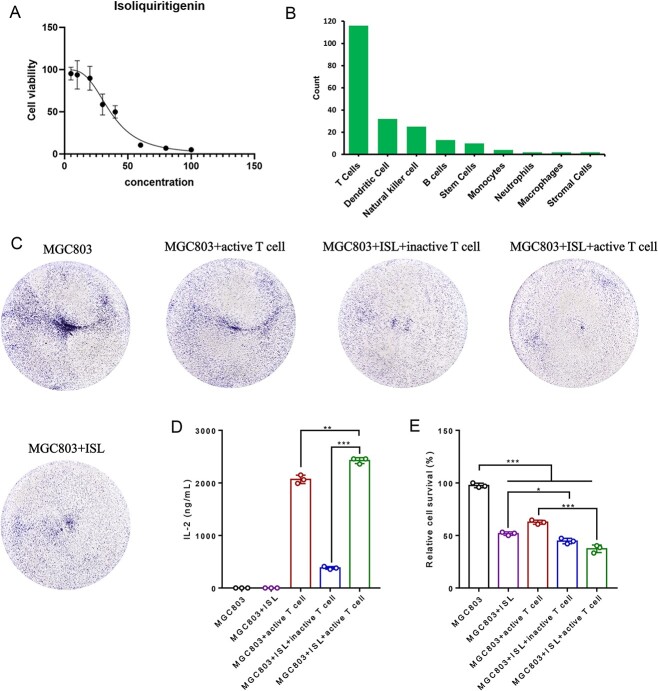
ISL enhances the viability of T cells and inhibits the proliferation of cancer cells. (**A**) The proliferation of MGC803 cells was assayed by CCK-8 assay after 24 h of treatment with ISL. (**B**) The number distribution of immune cells that significantly associated with ISL. (**C**) MGC803 cells, pretreated with ISL for 24 h, were co-cultured with activated Jurkat T cells and subsequently stained with crystal violet for imaging. (**D**) IL-2 levels in the supernatant of (C) were measured by ELISA kits (*n* = 3 replicates). (**E**) Crystal violet was dissolved in acetic acid and measured at 595 nm for cell viability detection of MGC803 cells (*n* = 3 replicates). Statistical significance: ^*^P < 0.05, ^*^^*^P < 0.01 and ^*^^*^^*^P < 0.001.

**Figure 7:**
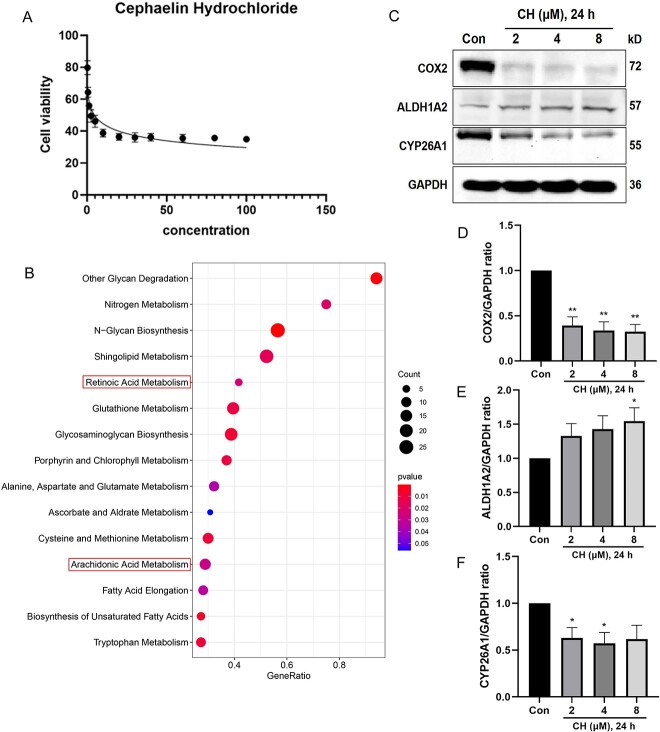
CH inhibits LGG proliferation and regulates arachidonic acid metabolism and retinoic acid metabolism. (**A**) Proliferation of SW1783 cells was detected by CCK-8 after treatment with CH 24 h. (**B**) The top 20% metabolic pathway that CH enriched. (**C**) Representative western blot bands of CYP26A1, ALDH1A2 and COX2. SW1783 cells were treated with various concentrations of CH for 24 h. (**D**–**F**) Quantitative analysis of COX2, ALDH1A2 and CYP26A1. Data are expressed as means ± SEM (*n* = 3). ^*^P < 0.05, ^*^^*^P < 0.01.

The effects of ISL and CH on MGC803 (STAD cells) and SW1783 cells (LGG cells) were assayed using the CCK-8 assay. The IC_50_ value of ISL against MGC803 cells was 36.19 μM (Figure 6A), indicating that ISL did not exert potent cytotoxic effects, which might means that ISL requires the involvement of immune cells to exert its anti-tumor effect. The IC_50_ value of CH against SW1783 cells was 4.721 μM (Figure 7A), indicating potent growth-inhibitory effects against LGG.

### ISL promotes the activation of T cell to suppress STAD

To investigate the specific immunomodulatory properties of ISL, the types of immune cells represented by the significantly associated ISs were counted (Figure 6B). The highest number of ISs associated with ISL was T cell status-related signatures, which suggested that ISL exerts anti-cancer effects by modulating T cells.

To investigate whether ISL exerts treatment effects on STAD cells by modulating T cells, an *in vitro* co-culture model comprising STAD and activated Jurkat cells was established. IL-2, a marker factor for activated T cells, was used to monitor T cell activation and proliferation. The results showed that the level of IL-2 in the supernatant was significantly increased upon treatment with ISL (Figure 6C and D). This indicated that ISL promoted the activation of Jurkat T cells and the release of IL-2. In addition, ISL-treated T cells exerted potent growth-inhibitory effects against MGC803 cells *in vitro* (Figure 6C and E). This indicated that activated Jurkat T cells potentiated the anti-cancer effects of ISL. Hence, ISL can exert growth-inhibitory effects against STAD by promoting the activation of T cells.

### CH is involved in the regulation of retinoic acid metabolism and arachidonic acid metabolism in LGG

To investigate the CH-modulated metabolic pathways, the pathways in which CH-related genes enriched were examined. The top 20% of metabolic pathways are shown in bubble diagrams (Figure 7B). Literature review suggested that arachidonic acid metabolism and retinoic acid metabolism play an essential role in the development of cancer [65–68]. Hence, this study focused on the proteins involved in these two pathways. CYP26 family enzymes and retinoic acid dehydrogenase (ALDH) are key enzymes for retinoic acid metabolism [69]. Meanwhile, the arachidonic acid pathway-metabolizing enzymes cyclooxygenases 2 (COX2) in arachidonic acid metabolism has also been considered as therapeutic targets for human cancer [66].

As shown in Figure 7C, CH treatment up-regulated ALDH1A2 expression and down-regulated CYP26A1 expression in MGC803 cells. This means that CH promoted the production of retinoic acid dehydrogenase, inhibited the production of CYP26 family enzymes and promoted retinoic acid accumulation, which could regulate the cell cycle to stop proliferation. The absence of retinoic acid signaling is associated with dedifferentiation and tumor development [70]. Therefore, CH inhibits MGC803 cell proliferation by inducing the accumulation of retinoic acid. In addition, COX2 was down-regulated, which is associated with inflammatory processes [71, 72]. The aberrant arachidonic acid metabolism observed in cancer cells is usually accompanied by an inflammatory state and a sustained increase in COX expression [65, 73]. It has been shown that COX2 knockdown or its inhibitor can suppress tumorigenesis, growth and progression [74].

## DISCUSSION

Traditional herbal systems have been an integral part of human history with repeated trials being performed on human subjects over thousands of years. In addition, herbal medicines play an essential role in the human health security of the general population [75]. Herbal ingredients can exert anti-cancer effects by inhibiting proliferation, regulating the immune microenvironment, modulating metabolism and reversing drug resistance, which can be attributed to their multiple targets, diverse biological activities and novel and diverse structures [75–77]. However, drug discovery from herbal medicine based on experiments is time consuming, expensive and laborious process [78, 79]. Therefore, effective methods are needed to improve traditional drug discovery. The increase in the amount of omics data in recent years has provided opportunities for the computational prediction of anti-cancer drugs and improved the efficiency of drug discovery [80]. Wang *et al*. proposed a strategy for high-throughput screening based on genetic markers of tumor immunophenotypes to discover immunotherapeutic compounds [22]. Pankaj Goswami *et al.* developed a novel drug interaction scoring algorithm to predict drug interaction effects in diffuse large B-cell lymphoma [81]. Cheng *et al*. proposed a network-based inference method to infer new targets for known drugs [82]. But, how to quantify the contribution of the specific pathways to the OCE of a drug has always been a challenging issue.

To address this issue, this study proposed a new computational framework COIMMR to quantify the contribution of the regulatory effects of herbal ingredients on the immune microenvironment and metabolic reprogramming to their anti-human cancer activities. In this study, a comprehensive data analysis of multiple cancers and herbal ingredients was performed. First, 30 human cancer data were obtained from TCGA, while 496 herbal ingredients data were obtained from ITCM. Next, high-quality ISs and MSs were collected and the NES values of each signature for each human cancer and herbal ingredient were calculated, and the immunological landscape and metabolic landscape were constructed. Finally, the contributions of herbal ingredients in their anti-human cancer activity through modulation of the immune microenvironment and metabolic reprogramming were quantified. To demonstrate the power of the developed method, *in vitro* experiments were performed with two representative cancers. ISL was identified to specifically target the T cells in STAD, while CH was identified to specifically target arachidonic acid metabolism and retinoic acid metabolism in LGG.

Compared with existing approaches like LINCS1000 [83, 84], our computational model has obvious differences and advantages. The prominent difference between our present work and LINCS1000 is that COIMMR could reveal the contribution of herbal ingredients against human cancer via specific pathways or biological function. It is also the first computational model to quantify the contribution of specific pathways to the OCE for herbal ingredients, which can fill the gap of LINCS1000. Secondly, datasets used in our computational model were all herbal ingredients from Traditional Chinese Medicine (TCM), and LINCS1000 contains very limited herbal ingredients [85]. Hence, our computational model pays more attention on the herbal ingredients space and could makes up for the limited herbal ingredients in LINCS1000. Thirdly, LINCS1000 cannot screen drugs targeting specific pathways, while our method can screen herbal ingredients targeting specific biological functions [86]. Fourthly, COIMMR was based on the full-length transcriptomic sequence platform of herbal ingredients, which measured the expression level of all protein coding genes (>20 000). However, LINCS1000 project only measured the expression level of 978 genes [87]. Hence, we are more reliable in terms of the number of genes sequenced and the quality of the data. Overall, COIMMR has its unique contributions for drug discovery, especially for herbal ingredients, which could promote the intelligent development of TCM.

For our model application and future drug development, one of the advantages is that it could rapidly analyze vast datasets, swiftly screen candidate compounds from herbal ingredients for drug development and improve the efficiency of translational drug discovery, which greatly reduces both the time and financial resources. In addition, it may facilitate the development of personalized medicine by targeting patient-specific pathways. This enables the design of specific drugs based on an individual's genetic makeup, improving efficacy and minimizing side effects. Furthermore, it could help researchers uncover intricate relationships, facilitating the discovery of new drug targets and mechanisms.

However, this study has some limitations. The expression profiling data of ITCM are based on the MCF-7 cell line. Hence, we encourage users to use cancer-specific drug expression data in the future studies. In addition, we also encourage users to build the customized specific signatures with professional knowledge and combine the analysis results with those obtained from COIMMR to arrive at the most appropriate conclusion. In summary, this computational strategy can be applied to development of drug targeting specific biological pathways for various diseases using the gene expression profile data. The findings of this study will aid in the modernization of TCM.

**Key Points**

- We constructed the first computational framework, COIMMR, which reveals the contribution of herbal ingredients against human cancer via immune microenvironment and metabolic reprogramming.
- By using COIMMR algorithm, we found that most herbal ingredients exerted a higher modulatory effect on the immune microenvironment than on metabolic reprogramming for their therapeutic effects on human cancer, which was first revealed by this study.
- By applying COIMMR algorithm to two case studies to demonstrate its strong power, we identified ISL that specifically regulates the T cells in STAD and CH that specifically targets metabolic reprogramming in LGG. The *in silico* results were verified using *in vitro* experiments.

## Supplementary Material

Supplementary_file_bbad346

## Data Availability

The data of our work can be acquired from the Supplementary Materials uploaded with this article.

## Author Biographies

**Saisai Tian** is a lecturer at School of Pharmacy, Ningxia Medical University. He has also served as alecturer at School of Pharmacy, Second Military Medical University, and his research interest covers bioinformatics, cancer biology, and network pharmacy.

**Yanan Li** is a postgraduate student of Ningxia Medical University, and her research interest covers cancer biology and network pharmacy.

**Jia Xu** is a postgraduate student of Henan University, and her research interest covers bioinformatics and network pharmacy.

**Lijun Zhang** is an associate professor at the Institute of Interdisciplinary Integrative Medicine Research, Shanghai University of Traditional Chinese Medicine, and her research interest is anti-tumor mechanism of traditional Chinese medicine.

**Jinbo Zhang** is an MPhil at the School of Pharmacy, Second Military Medical University and served as a pharmacist of Department of Pharmacy, Tianjin Rehabilitation Center of Joint Logistics Support Force. His research interest covers network pharmacy and natural products.

**Jinyuan Lu** is a postgraduate student at the School of Pharmacy, Anhui University of Chinese Medicine, and his research interest covers network pharmacy.

**Xike Xu** is a professor at the School of Pharmacy, Second Military Medical University, and his research interest covers network pharmacy.

**Xin Luan** is the director of Systems Pharmacology ResearchCenter of TCM, Institute of Interdisciplinary Integrative Medicine Research, Shanghai University of Traditional Chinese Medicine. His current research focuses on the discovery and novel application of anti-cancer compounds from traditional Chinese medicine.

**Jing Zhao** is a professor at the Institute of Interdisciplinary Integrative Medicine Research, Shanghai University of Traditional Chinese Medicine. Her current research focus on bioinformatics, cancer biology and network pharmacy.

**Weidong Zhang** is a professor at Ningxia Medical University, Second Military Medical University' Shanghai University of Traditional Chinese Medicine and Institute of Medicinal Plant DevelopmentChinese Academy of Medical Sciences and Peking Union Medical College. His major research interest areas are pharmacodynamic substance basis research of natural products and medicinal chemistry.
